# Impacts of Soil and Water Conservation Structures on Selected Soil Physicochemical Parameters in Wali Micro-Watershed Ambo District, Central Ethiopia

**DOI:** 10.1155/sci5/1465657

**Published:** 2025-05-04

**Authors:** Tesfaye Busa, Bayisa Duressa, Tena Regasa, Eve Bohnett, Siraj Mammo

**Affiliations:** ^1^College of Natural and Computational Science, Ambo University, Ambo, Ethiopia; ^2^School of Natural Resources, Ambo University, Ambo, Ethiopia; ^3^College of Natural and Computational Science, Wollega University, Nekemte, Ethiopia; ^4^Department of Landscape Architecture, College of Design Construction and Planning, University of Florida, Gainesville, Florida, USA

**Keywords:** conserved-farmlands, non-conserved farmlands, soil and water conservation, soil properties, Wali micro-watershed

## Abstract

Land degradation poses a significant environmental challenge, leading to reduced soil fertility, agricultural production, and overall land productivity. To combat this issue, the Ethiopian government has implemented various soil and water conservation (SWC) strategies in rural areas over the last few decades. This study assessed the impact of these SWC structures on selected soil physicochemical properties in Wali micro-watershed. A purposive sampling technique was used to select the study district and watershed, and a systematic randomized complete block design was used to collect soil samples. Samples were taken from both farmlands conserved with various SWC practices, like soil bund, stone bund, terraces, fanyajuu, and bench terrace, all from nonconserved lands along the lower, middle, slope classes using an “X” sampling design. Eighteen composite soil samples for all three slope classes were collected in triplicate from topsoil (0–30-cm depth) for analysis. These samples were processed and analyzed following standard laboratory procedures in the Ambo University Chemistry Department. The study assessed soil bulk density, moisture, pH, cation exchange capacity, electrical conductivity, organic matter, organic carbon, total nitrogen, available phosphorus, available potassium, and other basic cations (sodium, calcium, and magnesium). The results showed that conserved farmlands had mean values of 1.26 g/cm^3^ for bulk density, 6.72% for moisture, 6.29 for pH, 4.32% for organic matter, 2.24% for organic carbon, 0.30% for total nitrogen, 53.02 cmol/kg for CEC, 37.28 dS/cm for electrical conductivity, 25.80 mg/kg for available phosphorus, and 0.91 mg/kg for available potassium. In contrast, nonconserved farmlands exhibited 1.37 g/cm^3^ for bulk density, 6.07% for moisture, 5.86 for pH, 3.12% for organic matter, 1.84% for organic carbon, 0.21% for total nitrogen, 46.6 cmol/kg for CEC, 37.23 dS/cm for electrical conductivity, 21.06 mg/kg for available phosphorus, and 0.63 mg/kg for available potassium. The study concluded that SWC practices significantly improved soil bulk density, moisture content, pH, organic carbon, organic matter; cation exchange capacity, total nitrogen, sodium, available phosphorus, and potassium. However, electrical conductivity, calcium, and magnesium showed no significant improvement (*p* < 0.05). These findings highlight the positive effects of SWC structures on soil quality, underscoring the importance of maintaining these practices for sustainable land management and advocating for their expansion to other watersheds.

## 1. Introduction

Soil erosion remains one of the most significant environmental challenges globally, affecting both developed and developing countries [1]. The accelerated loss of topsoil, particularly from agricultural lands, has long been recognized as a critical threat to the world's soil resources [2]. Water-induced soil degradation, a major contributor to soil degradation [3], is increasingly recognized for its adverse impacts on agricultural production, soil fertility, and food security [4]. Projections indicate that over 90% of the Earth's soils could become degraded by 2050; 33% already degraded [5]. Annually, around 75 billion tons of fertile soil is eroded globally, resulting in the loss of about 12 million hectares of land [6].

Soil erosion is a global problem, which is critical particularly acute in rural areas of developing countries [7, 8], where land degradation poses major threats to ecosystem services, biodiversity conservation, food security, and the sustainability of livelihoods [9, 10]. In Ethiopia, land degradation significantly undermines agricultural productivity, leading to a reduction in crop yield and economic losses [11–14]. Effective conservation measures are crucial for mitigating soil degradation, improving soil health, and ensuring water availability [15, 16].

Water erosion, a primary driver of land degradation, results in both on-site and off-site effects, including infrastructure damage and water quality deterioration [17–23]. It accounts for about 80% of agricultural land degradation worldwide [5, 24]. In Ethiopia, soil erosion has become a primary cause of declining crop production and landscape degradation [25].

Topsoil depth and quality are critical factors in determining the extent of erosion on agricultural lands [26]. According to [27], the lack of appropriate soil and water conservation (SWC) exacerbates soil nutrient depletion, negatively impacting soil productivity and then household food security.

The Ethiopian highlands are particularly vulnerable, experiencing severe land degradation, soil erosion, and deforestation, which threaten natural resource conservation, crop productivity, and food security [28, 29]. Annually, Ethiopia loses about 42 Mg ha^−1^ yr^−1^ of soil [28, 30], with the highlands suffering an estimated 1.5 million tons of soil loss [31]. The degradation is even more pronounced on steep slopes and areas with sparse vegetation cover [32].

Addressing natural resource degradation is imperative for enhancing crop yields and improving the livelihoods of rural households in Ethiopia [33]. SWC is a sustainable, poverty-oriented resource management strategy that aims to preserve and enhance the productive capacity of land by preventing erosion, improving water retention, and maintaining soil fertility [34]. According to the sustainable land management (SLM) project hub [35], natural SWC activities at the local level help maintain or enhance the productive capacity of land, including soil, water, and vegetation, in areas prone to degradation through the prevention or reduction of erosion, compaction, salinity, and by conserving water and maintaining or improving soil fertility.

SWC is an integral component of watershed management, with numerous studies demonstrating its positive effects on soil properties, such as curbing soil loss, enhancing moisture retention, increasing organic matter, improving fertility status, reducing gully formation, and improving other soil properties like cation exchange capacity (CEC), nitrogen, phosphorus, and potassium [32, 36–41]. The introduction of SWC initiatives in Ethiopia began in the late 1990s, primarily through collaborations with SLM and GIZ (German led international project). Consequently, the Ethiopian government has initiated various SWC measures and large-scale rehabilitation projects across the nation to combat ongoing soil degradation. Each year, farmers across different regions engage in SWC initiatives to minimize sedimentation, reduce soil erosion and runoff speed, capture sediment and nutrients, improve downstream water quality, and enhance land productivity [39].

Despite these efforts, many SWC practices have been implemented through top-down approaches, often lacking appropriate follow-up, and with a predominant focus on physical measures, which has led to limitations in their overall effectiveness. Therefore, this study aimed to evaluate the impact of SWC practices on specific soil physicochemical characteristics in the Wali micro-watershed of the Ambo district, West Shoa, Ethiopia.

## 2. Materials and Methods

### 2.1. Description of the Study Area

#### 2.1.1. Location

**Figure 1:**
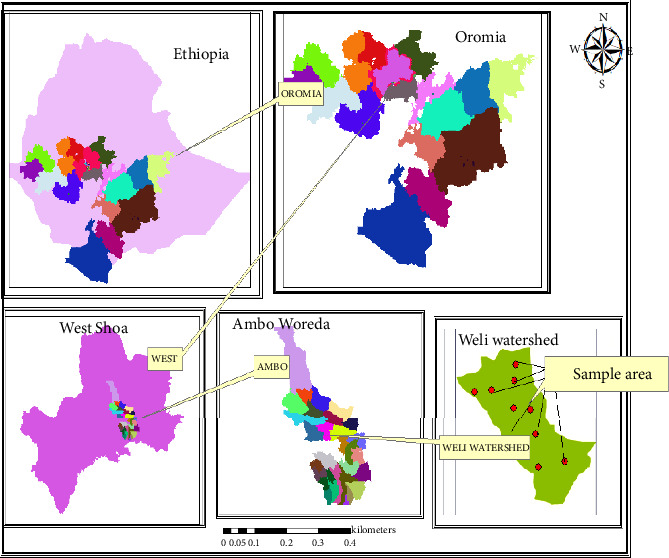
Geographic location of the study area in Ambo district, West Shoa zone, Oromia, Ethiopia.

The study was conducted in Ambo district, located within West Shoa Zone, Oromia, Ethiopia (Figure 1). Ambo serves as the district's capital and is situated 114 km to the west of Addis Ababa. Geographically, the district spans latitudes 8°47′N–9°21′N and longitudes 33°3′E–37°32′E, covering a total area of 83,598.69 ha [42]. T is bordered by Elfeta and Gindeberet districts to the north, Wonchi district to the southwest, Dendi district to the east, Toke Kutaye district to the west, and Midakegn district to the northwest.

The Wali micro-watershed, part of the West Shoa Zone Administration, is located in the Ambo district, south of Ambo town within the Abebe Doyo kebele of the Dabis major watershed. It lies between latitudes 8°47′N and 9°21′N and longitudes 37°32′E and 38°3′E, acting as a tributary of the Abay River. The study area encompasses approximately 356 ha, with land use distributed as follows: 187 ha of agricultural land, 78 ha of grazing land, 8 ha of forest and bushland, and 83 ha of homestead.

Topographically, the Wali micro-watershed features diverse terrain, comprising 8% undulating, 1% flat, 13% rolling, 47% hilly, and 31% steep and mountainous areas. The altitude ranges from 2300 to 2600 m above sea level (m.a.s.l.) (Ambo District Agriculture and Natural Resources office) [42]. Located 4.5 km from Ambo town, the area exhibits agro-ecological features with 68% classified as midland and 32% as highland [42].

#### 2.1.2. Socio-Economic Data

According to the Ambo District Finance and Economic Cooperation Office [43]; the district's population totals 113,087, with an equal gender distribution of 56,560 males and 56,560 females. The district's economy predominantly revolves around farming and livestock rearing, activities practiced consistently across various agro-ecological zones (Ambo District Agriculture and Natural Resources office) [42]. The principal crops cultivated include teff, wheat, barley, Niger seed, beans, peas, maize, and sorghum. Additionally, cash crops such as enset, tomatoes, potatoes, onions, and sugarcane are grown in significant quantities (Ambo District Agriculture and Natural Resources office) [42]. Livestock farming is substantial, with a total livestock population of 407,696, comprising 42.45% cattle, 25.15% sheep and goats, 23.48% poultry, and 8.92% pack animals (donkeys, mules, and horses).

#### 2.1.3. Climate and Topography

The climate of the Ambo district is characterized by a dominant midland climate, with a primary rainy season from June to September, peaking in July and August. The dry season extends from October to May, with occasional rains between February and April. The district's mean monthly rainfall ranges from 500 mm to 2700 mm, averaging 1200 mm over the last 12 years (Ambo Agricultural Research Center Meteorology Station, 2009–2020). Topographically, the district exhibits significant elevation diversity, with altitudes ranging from 1380 to 3030 m above sea level.

#### 2.1.4. Land Use

Arable land scarcity, driven by population pressures, is a critical issue in the study area [42]. This scarcity has led to continuous cultivation, resulting in soil fertility depletion and increased erosion. Rain-fed subsistence farming and animal husbandry remain the predominant agricultural practices.

#### 2.1.5. Vegetation

The area's vegetation is predominantly shrublands, interspersed with grasses and smaller plants. *Eucalyptus*, an exotic species known for its rapid growth and minimal management requirements, is widely planted around homesteads, farm fields, and along boundaries as windbreaks [44]. Despite its economic value-providing fuel wood, charcoal, construction materials, and timber, there is limited awareness among farmers about its negative impacts on soil fertility and water resources. The extensive planting of eucalyptus has led to the replacement of indigenous tree species, contributing to deforestation, as observed during field reconnaissance.

#### 2.1.6. Soil and Water Conservation Measures

In the study area, a variety of SWC measures have been implemented in the study area, tailored to the specific land use systems. These measures are crucial in mitigating land degradation, particularly in hilly and mountainous regions. Predominant SWC techniques include contour farming, soil bunds, stone bunds, fanyajuu terraces, bench terraces, check dams, and other moisture retention structures. Despite the widespread adoption of these practices, their impact on soil properties, fertility, productivity, and water resources remains under-researched.

### 2.2. Methods

#### 2.2.1. Reconnaissance Survey and Research Design

A reconnaissance survey was conducted to identify representative watersheds and establish appropriate sampling sites in both conserved and nonconserved farmlands. This preliminary survey was essential for selecting suitable micro-watersheds for comparison and identifying key landscape positions for sampling. Judgment sampling was employed to select representative sites in both soil conservation-treated (conserved) and untreated (nonconserved) micro-watersheds. This approach ensured that both treatment conditions were well represented in various landscape positions, enabling the effective comparison of soil properties across different management practices.

A randomized complete block design (RCBD) was used to structure the research design and facilitate soil sampling from conserved and nonconserved farmlands with SWC interventions.

#### 2.2.2. Sample Site Selection and Soil Sampling

The study district and watershed area were purposively selected due to the presence of SWC practices in the area, whereas soil sampling was conducted randomly along the micro-watershed. The watershed was selected due to the fact that SWC practices started earlier in the study area. Composite soil samples, representing the variability of each site, were collected in triplicate from a depth of 0–30 cm across different slope classes. Bulk density (BD) was measured using a core sampler, while a spiral auger was used for other soil samples. The experimental plots for both treatment types (nonconserved and conserved farmlands) were replicated three times to ensure reliable results. These plots were systematically selected based on slope gradients to provide a comprehensive understanding of soil conditions across various landscape positions.

The study area was divided into three distinct slope classes for soil sampling: upper slopes (> 30%), middle slopes (15%–30%), and lower slopes (< 15%). Soil samples were collected from each of these slope classes in the micro-watershed to account for potential variations in soil properties related to topography. A total of 18 composite soil samples, 9 from conserved farmlands and 9 from nonconserved farmlands, were collected during the dry season to avoid seasonal fluctuations in soil moisture. The samples were thoroughly mixed in a bucket to form composite samples, ensuring they were representative of the overall soil conditions at each site. Sub-samples of the mixed composite samples, each weighing approximately 1 kg, were then carefully labeled and transported to the Ambo University Chemistry Department laboratory for detailed analysis.

#### 2.2.3. Soil Laboratory Analysis

Soil analyses were conducted in the Chemistry and Soil Science Laboratories at Ambo University to assess both physical and chemical properties.

##### 2.2.3.1. Soil Physical Analysis

Soil BD was measured using undisturbed soil samples collected with a core sampler, following the procedure outlined by previous methods [45]. After drying the samples at 105°C for 24 h, the BD was calculated from the difference between the oven-dried weight of the soil and the volume of the core sampler, as described in equation (1):

(1) $$\begin{array}{c} \text{BD}\left(\frac{g}{{\text{cm}}^{3}}\right)=\frac{m}{V}, \end{array}$$

where *m* is the mass of the oven dry soil (g) and *V* is the volume of the core sampler.

In addition, soil water characteristics were estimated, including the field capacity and permanent wilting point, using a soil water characteristics calculator [16]. This allowed for the computation of the moisture content at these critical thresholds, as outlined in equation (2), where *wi* is the initial weight of the soil (g), and *wf* is the final weight of the soil after oven dry (g)

(2) $$\begin{array}{c} \text{Wt}.\%=\frac{wi-wf\times 100}{wf}. \end{array}$$

The soil moisture content (SMC) was determined using the gravimetric method. A 10-g soil sample was oven-dried at 105°C for 24 h to remove moisture. The moisture content was calculated by equation (3)

(3) $$\begin{array}{c} \text{Moisture content }\left(wt\%\right)=\frac{\text{wet soil weight}-\text{oven dry soil weight}\times 100}{\text{oven dry oven dry soil weight}}. \end{array}$$

##### 2.2.3.2. Soil Chemical Properties

Soil pH was measured using a pH meter in the supernatant suspension prepared with a 1:2.5 soil-to-water ratio, as described by [46]. Electrical conductivity (EC) was determined using a conductivity meter [47], allowing for the assessment of soil salinity.

Total nitrogen (TN) was quantified using the Kjeldahl method [45], which involves the oxidation of organic matter in a concentrated sulfuric acid solution (0.1 N H_2_SO_4_). The organic nitrogenous compounds were converted to ammonium sulfate during oxidation [48]. The ammonium ion (NH_4_^+^) released during distillation with sodium hydroxide (NaOH) was absorbed in boric acid and back-titrated with 0.02 N sulfuric acid (H_2_SO_4_). The available phosphorus was analyzed using the Olsen method [49], which employs sodium bicarbonate (0.5 M NaHCO_3_) as an extraction solution. Soil organic carbon (SOC) was determined using the wet digestion method [50]. Soil organic matter (SOM) was calculated as the product of SOC and a factor of 1.724 based on the assumption that organic matter consists of 58% carbon.

CEC was determined using 1 M ammonium acetate (NH_4_OAc) to saturate the soil samples at pH 7 [51]. The exchangeable bases were extracted using excess ammonium acetate, and the concentrations of sodium (Na) and potassium (K) were measured using a flame photometer. Calcium (Ca) and magnesium (Mg) concentrations were determined using an atomic absorption spectrophotometer (AAS) [51].

#### 2.2.4. Statistical Analysis

Data analysis was conducted using SPSS (Statistical Product and Service Solutions) version 28.0, formerly known as the Statistical Package for the Social Sciences. Prior to analysis, data were tested for normality to ensure the validity of parametric tests. The effect of SWC practices, as well as the slope gradient, on the physicochemical properties of the soil, was assessed using one-way analysis of variance (ANOVA). Tukey's range test (TRT) [52] was used for post hoc pairwise comparisons when significant differences were detected. A significance level of *p* ≤ 0.05 was used for all statistical tests.

## 3. Results and Discussion

### 3.1. Effect of SWC on Soil Physical Properties

#### 3.1.1. Soil Bulk Density

Soil BD is a critical indicator of soil compaction and soil health, which influences various soil properties such as root growth, available water capacity, soil porosity, infiltration, nutrient availability, and microbial activity [53]. In this study, soil BD was significantly (*p* < 0.05) affected by different SWC practices. The highest mean soil BD (1.43 ± 0.009 g/cm^3^) was recorded in the upper slope of nonconserved farmlands, while the middle and lower slopes of nonconserved lands recorded average values of 1.36 ± 0.006 g/cm^3^ and 1.32 ± 0.006 g/cm^3^, respectively (Table 1). In contrast, conserved lands showed lower values, with an average BD of 1.28 ± 0.006 g/cm^3^ on the upper slope and 1.25 ± 0.009 g/cm^3^ on the lower slope of the conserved farmlands.

These results align with previous studies [36, 54, 55], who reported that SWC practices generally lower soil BD due to the increase in organic matter from decomposing plant biomass and residues. The reduced BD observed in SWC-treated farmlands is indicative of improved soil health, as higher organic matter content enhances soil structure and porosity, which in turn reduces compaction [54]. The absence of SWC structures in nonconserved farmlands leads to higher BD, which is often associated with soil degradation and reduced soil fertility. SWC practices, including terraces and bunds, reduce slope gradients, slow runoff, and facilitate organic matter accumulation, thereby improving soil structure [56]. Moreover, reduced soil BD on conserved farmlands is related to improved soil structure and porosity as justified by various scientific studies.

#### 3.1.2. Soil Moisture Content (%MC)

SMC is a key parameter that influences the soil's physical properties and overall fertility. SMC is generally expressed as the ratio of the mass of water present in a soil sample to the mass of the sample after it has been dried at 105°C to a constant weight [57, 58]. In this study, soil moisture was significantly (*p* < 0.05) higher in farmlands treated with SWC measures compared to that in nonconserved lands. The average moisture content was 6.72 ± 0.223% in the conserved fields, compared to 6.07 ± 0.065% in the nonconserved fields (Table 1). The SWC measures improve the moisture of the soil and impound excess water through their influences on infiltration by increasing surface roughness and increasing the time of concentration [59].

The SMC values on nonconserved lands followed a clear trend of increasing moisture content from the upper slope (5.84 ± 0.047%) to the lower slope (6.27 ± 0.032%), with the middle slope recording 6.11 ± 0.017%. This trend is consistent with previous studies [38], which observed higher moisture accumulation at lower slope positions due to a reduced runoff and gravitational water movement. The lower slopes typically accumulate water from higher positions, creating concave areas that retain moisture more effectively.

In contrast, conserved lands demonstrated a marked improvement in moisture retention, with mean SMC values of 6.21 ± 0.062%, 6.52 ± 0.255%, and 7.45 ± 0.375% on the upper, middle, and lower slopes, respectively. The higher moisture content in conserved lands can be attributed to the effect of SWC measures such as terraces, bunds, and vegetation strips, which increase surface roughness, reduce runoff, and enhance water infiltration [60]. The reduced slope length in conserved lands allows for more time for water to infiltrate and be retained in the soil, as noted by Zeleke et al. [61].

These findings are consistent with the work of Chala et al. [54] who reported that SWC practices significantly improved the moisture-holding capacity of soils on degraded lands, particularly in areas with steeper slope gradients. Such improvements are critical in regions facing water scarcity, as SWC measures can significantly enhance soil moisture availability and, consequently, agricultural productivity.

Overall, these results emphasize the importance of SWC practices in improving soil moisture retention, especially in arid and semi-arid regions where water scarcity can limit agricultural productivity. Future research should focus on long-term monitoring to better understand the enduring effects of SWC practices on soil moisture dynamics over multiple growing seasons.

The results align closely with the findings of [54, 55], which demonstrated that farmland treated with SWC measures consistently exhibits higher SMC compared to untreated land. Similarly, the findings corroborate those of [32], who reported enhanced soil moisture levels in conserved farmland. This increased moisture retention is a positive indicator for improving crop yields, as soil moisture is critical for nutrient absorption and overall plant health. Consistent with these observations, Erkossa et al. [38] highlighted that plots treated with soil bund SWC structures exhibited significantly higher moisture content across different soil layers (0–30 cm and 30–60 cm) compared to untreated control plots. These findings underscore the importance of SWC practices in enhancing soil moisture and optimizing agricultural productivity.

### 3.2. Effect of SWC on Soil Chemical Properties

#### 3.2.1. Soil pH

Soil pH is a critical chemical property that influences various soil processes, including nutrient availability, toxicity, microbial activity, and root growth [62]. The measured mean pH values for conserved and nonconserved farmlands showed statistically significant differences (*p* < 0.05). Conserved farmlands exhibited mean pH values of 6.05 ± 0.029, 6.38 ± 0.015, and 6.45 ± 0.003 across the upper, middle, and lower slope classes, respectively, with an overall mean of 6.29 ± 0.062 (Table 2). This was notably higher than the pH values observed in nonconserved farmlands, which were 5.6 ± 0.006, 5.84 ± 0.104, and 6.13 ± 0.015 for the respective slope classes.

The higher soil pH values in conserved lands may be attributed to SWC practices, which enhance the retention of basic cations and reduce soil acidity. This aligns with findings by previous research [36, 37], which demonstrated increased pH in soils conserved with SWC interventions such as fanyajuu structures. Conversely, these results contradict with Dagnachew et al. [63], who reported a decreased pH in conserved watershed catchments under excessive rainfall conditions. The relatively lower pH in nonconserved lands is likely due to intensive cultivation, nutrient depletion, and the leaching of basic cations caused by soil erosion.

#### 3.2.2. EC

EC, an important indicator of soil health, reflects the salt content of the soil and its effects on plant nutrient availability, microbial activity, and other soil functions [62]. The mean EC values for conserved and nonconserved lands were 37.28 ± 1.798 dS/m and 37.23 ± 1.986 dS/m, respectively, showing no significant variation (*p* > 0.05) (Table 2). The slightly higher mean EC in conserved farmlands may be associated with the observed higher pH values, given the positive correlation between EC and pH [62]. Consistent with this observation, previous studies have reported marginal increases in EC on conserved farmlands compared to nonconserved areas [36, 37, 41]. Additionally, the gradual increase in EC from upper to lower slope classes in both farmland types can be attributed to the leaching of soluble salts, leading to their accumulation at lower slopes, a pattern corroborated by Tolesa et al. [32]. Previous studies also found this same result [32, 36]. Similar findings have shown slight EC increments on conserved farmlands, consistent with corresponding increases in soil pH [37]. In general, less fertile and nutrient-depleted soils tend to have lower EC due to reduced mineral content, while more productive soils exhibit higher conductivity and greater mineral concentrations.

Lands treated with SWC measures have also demonstrated significantly higher EC levels than untreated lands [32, 64]. The positive relationship between pH and EC further supports the hypothesis that conservation measures can enhance soil salt content, thereby contributing to improved soil chemical properties.

#### 3.2.3. TN

The laboratory analysis revealed significant differences (*p* < 0.05) in TN contents between conserved and nonconserved farmlands in the Wali micro-watershed. The mean TN value for conserved farmlands was 0.30 ± 0.018%, significantly higher than the 0.21 ± 0.047% recorded for nonconserved lands (Table 2). This finding aligns with previous research works of [32, 37, 40, 54, 65], which consistently demonstrated that farmlands treated with various SWC practices have higher TN levels. The enhanced TN content in conserved lands may be attributed to the integration of nitrogen-fixing leguminous plants as biological SWC techniques and the application of fertility-enhancing inputs such as manure and commercial fertilizers. Masha et al. [41] also reported elevated TN levels in lands conserved using level soil bunds, stone bunds, and fanyajuu systems.

A significant variation in TN was observed across slope classes (*p* < 0.05). In nonconserved farmlands, TN was higher at lower slopes (0.23 ± 0.006%) compared to that at upper slopes (0.18 ± 0.009%). Similarly, conserved farmlands showed a higher TN value at lower slopes (0.36 ± 0.006%) compared to that of upper slopes (0.24 ± 0.003%). This pattern likely results from the downward movement of organic materials and their subsequent accumulation at lower slopes, consistent with the finding of Tolesa et al. [32].

#### 3.2.4. CEC

CEC measures the soil's ability to retain essential nutrients (e.g. K^+^, NH_4_^+^, and Ca^2+^) in the plant-available form, serving as a critical indicator of soil fertility [66]. The implementation of SWC practices significantly (*p* < 0.05) influenced the CEC values in the study area (Table 2). Conserved farmlands recorded a higher mean CEC (53.02 ± 0.389 cmolc/kg) compared to nonconserved lands (46.6 ± 0.939 cmolc/kg).

The higher CEC observed in conserved lands may be attributed to the increased SOM content resulting from SWC practices. In the study by Challa et al. [54], this finding is consistent with previous research, which reported up to 27.93% higher CEC values in conserved lands compared to that of nonconserved ones [37, 54, 67]. Masha et al. [41] further highlighted that conservation structures such as level soil bunds and stone bunds significantly enhance CEC.

CEC values varied across slope classes, ranging from 51.9 ± 0.058 to 54.43 ± 0.203 cmolc/kg in conserved lands. Higher values were observed on gentle slopes compared to those on moderate slopes, indicating the influence of slope gradient on nutrient retention (Table 2).

#### 3.2.5. SOM

SOM is the organic component of soil, comprising plant and animal detritus at different stages of decomposition, cellular and tissue remnants of soil bacteria, and compounds synthesized by soil microbes. SOM is a vital component of soil, influencing its physical and chemical properties and contributing to ecological services essential for soil functions and quality [53]. The implementation of SWC practices significantly (*p* < 0.05) affected SOM levels (Table 2). Conserved farmlands exhibited higher SOM percentages along the slope gradient: 4.11 ± 0.014% at upper slopes, 4.28 ± 0.018% at middle slopes, and 4.57 ± 0.044% at lower slopes. In contrast, nonconserved farmlands recorded lower SOM values: 2.93 ± 0.046%, 3.10 ± 0.024%, and 3.35 ± 0.041% at upper, middle, and lower slopes, respectively.

The accumulation of biomass due to SWC interventions likely contributed to the observed SOM differences [56]. Previous studies have documented similar trends, comparing the relationship between terracing as SWC measure and soil fertility determinants [32, 40, 54].

The accumulation of biomass due to SWC interventions likely contributed to the observed SOM differences. Previous studies have documented similar trends, linking high SOM content to increased CEC levels [56, 62]. When comparing SOM content across slope classes, considerably elevated organic matter content was observed at the lower slopes of both conserved and nonconserved watersheds (Table 2). The downward movement of nutrients from upper to lower slopes further explains the higher SOM content at lower slopes, consistent with findings by Amare et al. [56] and Bekele et al. [68].

#### 3.2.6. SOC

SOC is a critical component of soil fertility, contributing approximately 50% of SOM [66]. The mean SOC value in farmlands treated with SWC measures was 2.24 ± 0.250%, significantly higher (*p* < 0.05) than the 1.84 ± 0.236% recorded for nonconserved lands (Table 2).

Higher SOC percentages were observed at lower slopes in both conserved and nonconserved farmlands. This pattern may be associated with the accumulation of organic residues and higher SOM at lower slopes. The lowest SOC mean value (1.46 ± 0.117%) was recorded in nonconserved lands, while the highest (3.2 ± 0.245%) was observed in conserved lands (Table 2).

These findings align with studies by Hailu et al. [36] and Dagnachew et al. [63], which reported that farmlands conserved with SWC practices, including fanyajuu, exhibited higher SOC levels than untreated plots. The enhanced SOC content underscores the importance of adopting SWC measures to improve soil fertility and carbon sequestration.

Moreover, the study by Belayneh et al. [39] reported significantly higher mean SOC values on farms treated with SWC practices compared to nonconserved lands. This increase is likely due to the higher SOM content on conserved lands, as SOM and SOC are positively correlated [53]. These findings are consistent with those of Dagnachew et al. [63] and Tolesa et al. [32], who also observed higher SOC levels on lands treated with various SWC measures compared to untreated farmlands. Wolka [59] further emphasized that SWC structures, such as soil bunds, help reduce surface runoff and soil erosion, thereby retaining more organic carbon and moisture in the soil [41]. Similarly, they found that lands treated with level soil bunds, stone bunds, and fanyajuu demonstrated considerably higher SOC levels than control plots. Additionally, slightly higher SOC values (2.19 ± 0.309%) were recorded at lower slope classes of untreated farmland compared to 1.87 ± 0.651% on upper slopes, a trend that aligns with findings of Teressa [67], Belayneh et al. [39], and Masha et al. [41].

#### 3.2.7. The Status of Available Phosphorus (Av.P)

Phosphorus (P) is an essential nutrient for plant growth and is present in various forms, including organic, mineral, and absorbed phosphorus [66]. Among these, available phosphorus (Av.P) is crucial for plant uptake. Laboratory analysis in this study revealed a statistically significant difference (*p* < 0.05) in Av.P levels between conserved and nonconserved farmlands. Conserved farmlands recorded a mean Av.P value of 25.80 ± 0.711 mg/kg, compared to 21.06 ± 0.808 mg/kg on nonconserved lands (Table 2).

The higher Av.P content on conserved farmlands is likely attributable to increased plant residues and organic matter accumulation in micro-watersheds treated with SWC measures. These findings align with previous studies, Hailu et al. [36] and Teressa [67], which reported elevated Av.P concentrations in plots with SWC structures like fanyajuu and soil bunds. Similar results were observed by Amare et al. [56], Belayneh et al. and Bekele et al. [39, 68], and Dagnachew et al. [63], who documented higher Av.P levels on conserved farmlands compared to untreated ones.

The mean values of Av.P, in relation to slope gradient, were examined as follows: 24.1 ± 0.377 mg/kg for upper farmlands, 24.87 ± 0.189 mg/kg for middle farmlands, and 28.43 ± 0.743 mg/kg for lower farmlands with conservation structures. On nonconserved farmlands, the variation was statistically insignificant (*p* > 0.089) but showed a gradual increase from upper to lower slopes, with mean values of 18.44 ± 0.124 mg/kg, 20.76 ± 0.091 mg/kg, and 23.99 ± 0.168 mg/kg, respectively. The slight increase at lower slopes may be due to fertilizer and manure runoff deposition, a finding consistent with studies by [32, 56].

#### 3.2.8. Available Potassium (Av.K)

The study found that SWC practices significantly influenced the available potassium (Av.K) levels across slope classes (Table 2). The detachment and transport of potassium from upper slopes and subsequent deposition at lower slopes resulted in slightly higher Av.K values at lower slope classes, a pattern similarly reported by Tolesa et al. [32]. The mean Av.K value on conserved farmlands was 0.91 ± 0.111%, compared to 0.63 ± 0.040% on nonconserved farmlands. The presence of various SWC structures appears to have created favorable conditions for nutrient retention, contributing to higher Av.K levels. These findings align with earlier studies by Hailu et al. [36] and Bekele et al. [37], who also reported higher potassium content on conserved plots.

#### 3.2.9. Exchangeable Bases (Na^+^, Ca^2+^, and Mg^2+^)

Exchangeable bases, including sodium (Na^+^), calcium (Ca^2+^), and magnesium (Mg^2+^), are critical indicators of soil fertility and contribute to the soil's CEC.

  Calcium (Ca^2+^): Although the mean value of exchangeable calcium was slightly higher on conserved farmlands, the difference was not statistically significant (*p* > 0.05). The recorded values on conserved slopes were 0.137 ± 0.009 cmol/kg, 0.22 ± 0.025 cmol/kg, and 0.22 ± 0.006 cmol/kg for upper, middle, and lower slope classes, respectively.
  Magnesium (Mg^2+^): The overall mean Mg^2+^ value was 0.064 ± 0.008 cmol/kg on conserved lands and 0.047 ± 0.049 cmol/kg on nonconserved lands, with no statistically significant differences. These results are consistent with findings by Hailu et al. [36] and Amare et al. [56], who also observed insignificant differences in exchangeable Ca^2+^ and Mg^2+^ between conserved and nonconserved lands.
  Sodium (Na^+^): No significant differences (*p* > 0.05) were observed in exchangeable sodium levels between conserved and nonconserved farmlands. Mean values were 2.48 ± 0.056 cmol/kg and 2.49 ± 0.050 cmol/kg, respectively. On nonconserved slopes, Na^+^ levels were 2.45 ± 0.092 cmol/kg (upper), 2.47 ± 0.070 cmol/kg (middle), and 2.55 ± 0.116 cmol/kg (lower). In conserved areas, the corresponding values were 2.33 ± 0.014 cmol/kg, 2.41 ± 0.032 cmol/kg, and 2.68 ± 0.055 cmol/kg, respectively (Table 3).

These findings collectively highlight the role of SWC practices in enhancing soil nutrient retention, particularly for potassium and phosphorus, while having a more limited impact on exchangeable bases.

## 4. Conclusions and Recommendations

Soil degradation, particularly through erosion, poses a significant threat to agricultural productivity, biodiversity, food security, and community livelihoods. Erosion-driven loss of fertile topsoil adversely impacts crop production, necessitating the implementation of SWC measures, including physical, biological, and agronomic interventions. Despite the adoption of various SWC practices in the Wali micro-watershed, Ambo, Oromia, Ethiopia, their impact on soil properties has not been extensively studied. This research aimed to assess the influence of SWC measures on selected soil physical and chemical properties in the micro-watershed.

The study involved the collection and analysis of 18 composite soil samples from conserved and nonconserved farmlands across lower, middle, and upper slope classes. Key soil physical properties (BD and moisture content) and chemical parameters (pH, EC, CEC, organic matter, organic carbon, TN, available phosphorus, available potassium, and exchangeable cations such as calcium, magnesium, and sodium) were analyzed following standard laboratory protocols.

The findings revealed significant differences in soil physical and chemical properties between conserved and nonconserved farmlands. Conserved farmlands exhibited superior soil characteristics, including higher moisture content, organic carbon, organic matter, CEC, TN, available phosphorus, and available potassium compared to nonconserved plots. These improvements suggest that the implementation of SWC measures has positively influenced soil fertility, nutrient availability, and productivity. However, no statistically significant differences (*p* > 0.05) were observed in certain chemical properties, including EC and basic cations such as calcium and magnesium.

Current SWC practices in the Wali micro-watershed are limited to physical measures such as soil bunds, terraces, contour farming, fanyajuu, and check dams. To further enhance soil quality and mitigate erosion, it is recommended that integrated SWC approaches combining physical, biological, and agronomic measures be introduced. A due consideration should be paid on biological SWC measures due to their feasibility, ease of implementation, and profound effect on soil moisture and fertility. There should be close monitoring and evaluation of these SWC by government. Moreover, community participation and stewardship among the local farmers and community should be enhanced by concerned bodies. Additionally, further research should be conducted to explore the long-term impacts of SWC practices on crop yield, soil fertility, water resources, and community livelihoods, as well as other soil properties not addressed in this study. These efforts are essential for sustainable watershed management and improved agricultural productivity in the region [43, 69].

## Figures and Tables

**Table 1:** Status of selected soil physical properties.

Variable	Slope classes	BD (g/cm^3^) Mean + SE	MC (%) Mean + SE
Conserved	Upper	1.28 ± 0.006^a^	6.21 ± 0.062^b^
	Middle	1.26 ± 0.009^b^	6.52 ± 0.255^b^
	Lower	1.25 ± 0.009^b^	7.45 ± 0.375^a^
	Overall mean	1.26 ± 0.006	6.72 ± 0.223
	*p*-value	< 0.0008	< 0.0004

Non-conserved	Upper	1.43 ± 0.009^a^	5.84 ± 0.047^c^
	Middle	1.36 ± 0.006^b^	6.11 ± 0.017^b^
	Lower	1.32 ± 0.006^b^	6.27 ± 0.032^a^
	Overall mean	1.37 ± 0.016	6.07 ± 0.065
	*p*-value	< 0.000	< 0.000

*Note:* The same letters along the column show non-significance among the slope gradient, while different letters show significant difference.

Abbreviations: BD = bulk density, MC = moisture content.

**Table 2:** Status of selected soil chemical properties.

Variable	Slope classes	pH Mean + SE	SOM (%) Mean + SE	SOC (%) Mean + SE	TN (%) Mean + SE	CEC (cmol/kg) Mean + SE	EC (dS/cm) Mean + SE	Av.P (mg/kg) Mean + SE	Av.K (mg/kg) Mean + SE
Conserved	Upper	6.05 ± 0.029^b^	4.11 ± 0.014^c^	1.75 ± 0.040^b^	0.24 ± 0.003^c^	51.9 ± 0.058^a^	30.4 ± 0.833^b^	24.1 ± 0.377^a^	0.6 ± 0.000^b^
	Middle	6.38 ± 0.015^a^	4.28 ± 0.018^b^	1.78 ± 0.025^b^	0.29 ± 0.003^b^	52.67 ± 0.088^a^	41.33 ± 1.462^a^	24.87 ± 0.189^a^	0.8 ± 0.067^b^
	Lower	6.45 ± 0.003^a^	4.57 ± 0.044^a^	3.2 ± 0.245^a^	0.36 ± 0.006^a^	54.43 ± 0.203^a^	40.1 ± 0.351^a^	28.43 ± 0.743^b^	1.33 ± 0.047^a^
	Over all mean	6.29 ± 0.062	4.32 ± 0.069	2.24 ± 0.250	0.30 ± 0.018	53.02 ± 0.389	37.28 ± 1.798	25.80 ± 0.711	0.91 ± 0.111
	*p*-value	< 0.0009	< 0.0002	< 0.001	< 0.000	< 0.005	> 0.07	< 0.023	< 0.000

Non-conserved	Upper	5.6 ± 0.006^b^	2.93 ± 0.046^c^	1.87 ± 0.651^b^	0.18 ± 0.009^b^	49.6 ± 0.265^a^	29.5 ± 0.289^b^	18.44 ± 0.124^c^	0.73 ± 0.044^a^
	Middle	5.84 ± 0.104^b^	3.10 ± 0.024^b^	1.46 ± 0.117^c^	0.22 ± 0.003^a^	43.93 ± 0.176^c^	39.6 ± 0.305^a^	20.76 ± 0.091^c^	0.59 ± 0.037^b^
	Lower	6.13 ± 0.015^a^	3.35 ± 0.041^a^	2.19 ± 0.309^a^	0.23 ± 0.006^a^	46.27 ± 1.538^b^	42.6 ± 0.208^a^	23.99 ± 0.168^a^	0.57 ± 0.088^b^
	Over all mean	5.86 ± 0.083	3.12 ± 0.063	1.84 ± 0.236	0.21 ± 0.047	46.6 ± 0.939	37.23 ± 1.986	21.06 ± 0.808	0.63 ± 0.040
	*p*-value	< 0.0001	< 0.028	< 0.000	< 0.002	< 0.000	> 0.364	> 0.089	< 0.000

*Note:* Av.P = available phosphorus, Av.K = available potassium. Similar letters along the column show no significance within slope classes, while different letters indicate significance difference.

Abbreviations: CEC = cation exchange capacity, EC = electrical conductivity, SOC = soil organic carbon, SOM = soil organic matter, TN = total nitrogen.

**Table 3:** Status of exchangeable bases of selected soil.

Variable	Slope classes	Na^+^ (cmol/kg) Mean + SE	Ca^+2^ (cmol/kg) Mean + SE	Mg^+2^ (cmol/kg) Mean + SE
Conserved	Upper	2.33 ± 0.014^c^	0.137 ± 0.009^b^	0.06 ± 0.006^b^
	Middle	2.41 ± 0.032^b^	0.22 ± 0.025^a^	0.09 ± 0.009^a^
	Lower	2.68 ± 0.055^a^	0.22 ± 0.006^a^	0.04 ± 0.006^b^
	Overall mean	2.48 ± 0.056	0.19 ± 0.016	0.064 ± 0.008
	*p*-value	> 0.072	> 0.065	> 0.34

Non-conserved	Upper	2.45 ± 0.092^b^	0.22 ± 0.012^a^	0.083 ± 0.003^a^
	Middle	2.47 ± 0.070^b^	0.21 ± 0.006^a^	0.04 ± 0.003^b^
	Lower	2.55 ± 0.116^a^	0.21 ± 0.006^a^	0.02 ± 0.147^b^
	Overall mean	2.49 ± 0.050	0.21 ± 0.005	0.047 ± 0.049
	*p*-value	> 0.45	> 0.57	> 0.47

*Note:* Mg = magnesium, Ca = calcium, Na = sodium. Similar letters along the column show no significance within slope classes, while different letters indicate significance difference.

## Data Availability

The data that support the findings of this study are available from the corresponding author upon reasonable request.
